# Profil épidémiologique des uvéites: à propos de 105 cas

**DOI:** 10.11604/pamj.2016.24.97.9188

**Published:** 2016-05-27

**Authors:** Abdoul Salam Youssoufou Souley, Hamed Ould Mohamed Abdellah, Mehdi Khmamouche, Alwan Alsubari Naji, El Ouatassi Narjis Fouad Elasri, Karim Reda, Abdelbarre Oubaaz

**Affiliations:** 1 Service d'Ophtalmologie à l'Hôpital Militaire d'Instruction Mohamed V de Rabat, Rabat, Maroc

**Keywords:** Uvéite, épidémiologie, étiologies, Uveitis, epidemiology, etiologies

## Abstract

La difficulté du diagnostic des uvéites réside dans la multiplicité des étiologies. La détermination des facteurs épidémiologiques des uvéites permettent une meilleure orientation diagnostique et facilitent leur prise en charge thérapeutique. C'une étude rétrospective s’étalant sur 4 ans de janvier 2012 à décembre 2015. Nous avons colligé 105 dossiers d'uvéite et étudié les aspects épidémiologiques, cliniques et étiologiques. L’âge moyen était de 42 ans, la tranche d’âge la plus touchée était de 40-50ans. Les hommes étaient plus atteints (57,14%) que les femmes (42,86%). L'uvéite était unilatérale dans 60,95% des cas. Nous avons noté une uvéite antérieure dans 35,24%, une uvéite intermédiaire dans 5,71%, une uvéite postérieure dans 10,48% et une panuvéite dans 48,57%. Les étiologies étaient dominées par la maladie de Behçet, la sarcoïdose, et la maladie de Vogt-Koyanagi-Harada. L'origine restait indéterminée dans 43,81% des cas. L’évolution était variable. Les uvéites sont des inflammations intraoculaires qui peuvent représenter une réelle menace pour la vision. La démarche permettant de découvrir la cause doit être codifiée et repose sur plusieurs étapes. Nos résultats sont relativement proches de ceux de la littérature. Les uvéites constituent une entité clinique où ophtalmologie et médecine interne se rejoignent. Le champ des causes et des investigations permettant le diagnostic est vaste voire illimité. Cette étude a permis de mettre en évidence les difficultés cliniques et de souligner les limites de notre formation sanitaire dans la prise en charge des uvéites.

## Abstract (en)

The multiplicity of causes of uveitis makes diagnosis difficult. Determining epidemiological factors associated with uveitis allows better diagnostic orientation and facilitates therapeutic management. This is a retrospective study spanning four years from January 2012 to December 2015. We collected 105 cases with uveitis and studied its epidemiological, clinical and etiological aspects. The average age was 42 years, the most affected age group was 40-50 years. Men were more affected (57.14%) than women (42.86%). Uveitis was unilateral in 60.95% of cases. Anterior uveitis was found in 35.24%, intermediate uveitis in 5.71%, posterior uveitis in 10.48% and panuveitis in 48.57%. Etiologies were dominated by Behçet's disease, sarcoidosis and Vogt-Koyanagi-Harada disease. The origin remained unknown in 43.81% of cases. The evolution was variable. Uveitis is an intraocular inflammation which may represent a real threat to vision. The approach to discover its cause needs to be codified and based on several steps. Our results are relatively close to those found in the literature. Uveitis is a clinical entity where ophthalmology and internal medicine meet. The field of its causes and investigations for the diagnosis is extensive or even unlimited. This study highlighted the clinical challenges and emphasized the limitations of our clinical training in the management of uveitis.

## Introduction

L'uvéite est un état inflammatoire de l'uvée composée de l'iris, le corps ciliaire et la choroïde. Cette inflammation peut s'inscrire dans le cadre d'une maladie systémique ou constituer une affection oculaire isolée. La détermination des facteurs épidémiologiques et étiologiques des uvéites permettent une meilleure orientation diagnostique et facilitent leur prise en charge thérapeutique, tel était notre objectif dans cette étude.

## Méthodes

Il s'agit d'une étude rétrospective s’étalant sur 4 ans de janvier 2012 à décembre 2015 à l'Hôpital Militaire d'Instruction Mohamed V de Rabat. Nous avons recensé, pour cette période, 105 dossiers d'uvéite et étudié les aspects épidémiologiques, cliniques et étiologiques. Nous avons inclus dans notre étude tous les cas d'uvéite hospitalisés dans notre service pendant la période indiquée ci-dessus de l’étude. Tous ces dossiers ont été exploités pour relever les différents paramètres étudiés. Sont exclus de notre travail: les uvéites vues en ambulatoire, les endophtalmies post traumatiques et post-chirurgicales, les uvéites phaco-antigéniques, les sclérites isolées, et les affections tumorales. Tous les patients ont bénéficié d'un examen ophtalmologique complet et une angiographie à la fluorescéine quand le siège de l'atteinte l'imposait. D'autres examens ophtalmologiques ont été réalisés quand l'orientation de l'examen clinique l'exigeait. Nous nous sommes basés pour le diagnostic de la forme anatomique de l'uvéite sur: la classification de l'international uveitis study group. Le diagnostic étiologique a été établi sur la base de l'anamnèse, l'examen ophtalmologique, de l'examen général; des examens paracliniques standards (NFS, avec VS, une CRP, une sérologie syphilis, une IDR a la tuberculine et une radiographie du thorax) et d'autres examens complémentaires ciblés en fonction de l’étiologie.

## Résultats

L’âge de nos patients varie entre 9 et 85 ans. Les patients âgés de 40 à 50 ans étaient majoritaires représentant 37,14% de notre série. Le sex ratio Homme/Femme est de 1,3/1. L'uvéite était unilatérale chez 60,95% des patients. Une BAV profonde < 1/10 est prédominante chez 38,09% avant le traitement. Les uvéites totales sont majoritaires avec 48,57% des cas, suivies des uvéites antérieures avec 35% puis postérieures et intermédiaires (Tableau 1). Les uvéites antérieures non granulomateuses sont prédominantes avec 66,66% des cas. La majorité des patients soit 85,71% présentaient une normotonie. Une étiologie a été déterminée chez 56,19% des cas. La principale étiologie retrouvée est la maladie de Behçet avec 20%, de la sarcoïdose avec 9,52% et enfin la maladie de Vogt-Koyanagi-Harada avec 7,61% (Tableau 2). La majorité des patients c'est à dire 52,39% présentent une amélioration nette, avec une acuité visuelle située entre 7/10 et 10/10 après le traitement.

**Tableau 1:** Répartition de l'uvéite selon le site anatomique

Site anatomique	Nombre de cas	Pourcentage
Uvéites antérieures	37	35,24%
Uvéites intermédiaires	06	5,71%
Uvéites postérieures	11	10,48%
Panuvéites	51	48,57%
Total	105	100%

**Tableau 2:** Répartition des uvéites en fonction des étiologies

Etiologies	Nombre de cas	pourcentage
Maladie de Behçet	21	20%
Sarcoïdose	10	9,52%
Maladie de Vogt Koyanagi Harada	08	7,61%
Herpès Simplex Virus	07	6,67%
Spondylarthrite ankylosante	04	3,81%
Tuberculose	02	1,90%
Arthrite idiopathique juvénile	01	0,95%
Syndrome TINU	01	0,95%
Virus Zona Varicelle	01	0,95%
Syphilis	01	0,95%
Fièvre boutonneuse méditerranéenne	01	0,95%
Infection loco-régionnale (sinusite)	01	0,95%
Toxoplasmose	01	0,95%
Indéterminée	46	43,81%
Total	105	100%

## Discussion

La distribution des types d'uvéites et de leurs étiologies dans une population donnée est fortement influencée par une variété de facteurs génétiques, géographiques, environnementaux et socioéconomiques. Dans les pays occidentaux et américains, l'incidence des uvéites serait 17 à 52 cas pour 100 000 habitants par an et leur prévalence de 38 à 204 cas pour 100 000 habitants [1, 2]. Si l'on considère l'ensemble des uvéites, toutes causes confondues, le sex-ratio est homogène dans la majorité des grandes séries [3]. Il varie entre 0,7 et 1,2. Il est de 1,3 dans notre étude. Il varie considérablement en fonction des étiologies. Les uvéites surviennent à tout âge avec un pic de fréquence chez les individus en âge de travailler. Dans notre série la tranche d’âge la plus touchée est de 40 à 50 ans et représente 37,14%. Nos résultats sont comparables à ceux de la littérature [3]. La grande majorité des séries rapportent des fréquences plus faibles d'uvéites chez les enfants et les personnes âgées [4, 5]. L’étude nord américaine montre une fréquence plus élevée chez les personnes âgées [1]. Les uvéites antérieures représentent 35,24%, les uvéites intermédiaires 5,71%, les uvéites postérieures 10,48% et les panuvéites 48,57%. Dans des études occidentales et américaines, les uvéites antérieures constituent le type anatomique prédominant 28,5% à 62% suivies des uvéites postérieures 13 à 26%, puis les uvéites intermédiaires et les panuvéites [2, 6]. La comparaison de nos résultats avec les autres études montre une grande diversité dans la répartition étiologique des uvéites d'une série à l'autre. Bodaghi et al. ont trouvé 28,5% d'uvéites antérieures, intermédiaires 15%, postérieures 21,6% et 35% de panuvéites [7] (Tableau 3). Les 3 principales étiologies d'uvéites chez nos patients, tout type confondu sont représentées par: la maladie de Behçet 20%, puis la sarcoïdose 9,52% et la Maladie de Vogt Koyanagi Harada 7,61%. Des fréquences similaires ont été rapportées dans plusieurs séries de proche et extrême orient, et les pays situés autour du bassin méditerranéen [8]. Chebil et al. ont trouvé la maladie de Behçet en premier 14,7% suivie de toxoplasmose 10,2% puis VKH 3,7% [9]. Lorsqu'il existe une orientation étiologique, les uvéites infectieuses sont majoritaires 30% à 50% des étiologies [10]. La principale faiblesse dans notre étude est le nombre restreint de malades; seules les uvéites graves ont été retenues dans l’étude. Les malades vus en ambulatoire n'ont pas été retenus dans la série.

**Tableau 3:** Fréquence des uvéites selon la localisation anatomique par rapport à la littérature.

	Bodaghi et coll.	Kazokoglu et coll.	Chebil et coll.	Notre étude
Pays	France	Turquie	Tunisie	Maroc
Nombre de patients	927	761	424	105
Uvéites antérieures	28,5%	52,5%	48%	35,24%
Uvéites intermédiaires	15%	6,7%	5%	5,71%
Uvéites postérieures	21,6%	12,7%	13,3%	10,48%
Panuvéites	35%	28,1%	33,6	48,57%

## Conclusion

Dans notre étude les étiologies les plus fréquentes d'uvéite étaient la maladie de Behçet, l'herpès simplex virus puis la sarcoïdose. La connaissance du profil épidémiologique dans une population donnée est une aide précieuse dans l'orientation de la démarche étiologique. La prise en charge doit être multidisciplinaire pour un meilleur rendement diagnostic et thérapeutique.

### Etat des connaissances sur le sujet

- Les étiologies des uvéites sont nombreuses et la prise en charge est multidisciplinaire;
- Dans la majorité des cas, l’étiologie est indéterminée.

### Contribution de notre étude à la connaissance

- Amélioration de la prise en charge et du suivi des patients présentant une uvéite;
- Très grande fréquence de la maladie de Behçet, de la sarcoïdose et la maladie Vogt Koyanagi Harada.
